# Histological and immunohistochemical features of the spleen in persistent polyclonal B-cell lymphocytosis closely mimic splenic B-cell lymphoma

**DOI:** 10.1186/1746-1596-7-107

**Published:** 2012-08-19

**Authors:** Ping Sun, Ridas Juskevicius

**Affiliations:** 1 Division of Hematopathology, Diagnostic Services of Manitoba, University of Manitoba, MS559S-820 Sherbrook Street, Winnipeg, R3A 1R9, MB, Canada; 2 Department of Pathology & Laboratory Medicine, Brody School of Medicine, East Carolina University, 600 Moye Blvd, Brody Medical Sciences Building 7S-18, Greenville, NC, 27858-4353, USA

**Keywords:** Persistent polyclonal B cell lymphocytosis, Splenomegaly, Lymphoma, Binucleated lymphocytes

## Abstract

Persistent polyclonal B-cell lymphocytosis (PPBL) is rare and intriguing hematological disorder predominantly reported in young to middle- aged smoking women. It is characterized by persistent moderate polyclonal B-cell lymphocytosis with circulating hallmark binucleated lymphocytes and elevated polyclonal serum IgM. Most patients have benign clinical course on long-term follow-up. Some pathologic features of PPBL may resemble malignant lymphoma, including morphology as well as frequent cytogenetic and molecular abnormalities. Significant symptomatic splenomegaly requiring splenectomy is very unusual for this disorder; therefore there is a lack of descriptions of the morphologic features of the spleen in the literature. We present here one of the first detailed descriptions of the morphologic and immunohistochemical features of the spleen from a young female with PPBL who developed massive splenomegaly during 6-year follow up. Splenectomy was performed for symptomatic relief and suspicion of malignant process. The morphological and immunohistochemical features of the spleen closely mimicked involvement by B-cell lymphoma, however there was no monotypic surface light chain restriction seen by flow cytometry and no clonal rearrangement of *IgH* gene was detected by molecular analysis. Evaluating a splenectomy sample in cases like this may present a diagnostic challenge to pathologists. Therefore, correlation with B cell clonality studies (by flow cytometry and molecular analysis), clinical findings and peripheral blood morphology searching for characteristic binucleated lymphocytes is essential to avoid misdiagnosing this benign process as B-cell lymphoma. We also present here a literature review on pathogenesis of PPBL.

**Virtual slides:**

The virtual slide(s) for this article can be found here: http://www.diagnosticpathology.diagnomx.eu/vs/5329558967545656

## Background

Persistent polyclonal B-cell lymphocytosis (PPBL) is a rare clinically benign lymphoproliferative disorder first described by Gordon et al. in 1982 [1]. To date, around 200 cases worldwide have been reported in literature. PPBL is characterized by chronic mild-to-moderate peripheral polyclonal lymphocytosis of B cell origin as evidenced by flow cytometry and variable number of atypical binucleated lymphocytes present on peripheral blood film. The total lymphocyte count is not always elevated in all patients. Due to the hallmark presence of binucleated lymphocytes, this entity is also called B-cell lymphocytosis with binucleated lymphocytes. The majority of patients also demonstrate polyclonal increase in serum IgM. It is most frequently reported in young or middle-aged female smokers [1-6]. Most patients present with mild nonspecific symptoms, such as weakness and fatigue. Mild splenomegaly is a single most frequently reported clinical finding, present in about 10% of patients according to the largest case series [2]. Massive splenomegaly has been only rarely reported in the literature. As most patients have an indolent clinical course and peripheral lymphocytosis is sometimes absent, this condition is likely under-recognized. PPBL resembles malignant lymphoproliferative disorder in many aspects, both morphologically and at the molecular/cytogenetic levels. In addition to the hallmark atypical lymphocytes, the bone marrow of these patients sometimes shows intrasinusoidal distribution of B lymphocytes resembling marrow involvement by splenic marginal zone lymphoma [7]. We here report histological and immunohistochemical features of the spleen in a young female with PPBL who underwent splenectomy due to progressive splenomegaly on her 6^th^ annual follow-up. Very recently, Italian investigators Del Giudice *et al.* described similar histopathological findings of the spleen in three patients with PPBL and progressive splenomegaly who underwent splenectomy [8]. PPBL can be further confused with a malignant lymphoproliferative disorder as PPBL is frequently associated with chromosomal abnormalities and multiple *BCL2/IG* gene rearrangements as seen in some lymphomas [9,10]. To avoid unnecessary aggressive treatment, it is important for pathologists to distinguish this clinically benign disorder from malignant lymphoproliferative disorders.

## Case presentation

The 38-year-old woman with a 20 years history of heavy smoking of 1 ½ packages of cigarettes per day was referred to QEII Health Science Centre, Halifax, Nova Scotia about 6 years ago for investigation of fatigue, frequent colds and persistent lymphocytosis for over 6 months. Her past medical history was unremarkable. The only positive finding on her physical examination was mild splenomegaly with spleen palpable 4 cm below left costal margin. Her initial complete blood cell count (CBC) showed isolated lymphocytosis of 10.2 x 10^9^/L with peripheral blood smears showing few atypical binucleated lymphocytes. Flow cytometry of her peripheral blood revealed an increase in polytypic CD19+/ CD20+/ CD5-/ CD10- B cells. Serum protein electrophoresis showed elevated polyclonal IgM at 9.6 g/L. The patient was diagnosed with PPBL and was closely monitored by her family physician and local oncologist. Over the following 6 years, she received no medical intervention. Several months prior to her splenectomy, she started complaining of increasing abdominal discomfort. Her spleen slowly increased in size and reached over 8 cm below left costal margin. She had no hepatomegaly or lymphadenopathy on CT imaging of the neck, chest, abdomen and pelvis. Her CBC remained unchanged with persistent peripheral lymphocytosis varying between 10 to 13 x 10^9^/L and showing variable numbers of characteristic binucleated lymphocytes. Repeated flow cytometry of peripheral blood just prior to splenectomy showed persistent increase in polytypic B cells. She subsequently underwent splenectomy for symptomatic relief as well as for suspicion of “malignant transformation”. Patient’s postoperative course was unremarkable requiring no further therapy. The patient continued heavy smoking throughout her years of follow-up.

## Materials and methods

### Histology and immunohistochemistry

Formalin-fixed, paraffin-embedded tissue sections were stained with hematoxylin and eosin (H&E) and Periodic Acid-Schiff (PAS). Immunohistochemistry was performed on 4-μm-thick sections prepared from formalin-fixed, paraffin-embedded tissue using an automated immunostainer (Bechmark XT, Ventana Medical Systems Inc., Tucson, AZ). The antigenic determinants and probes tested are listed in table 1.

**Table 1:** List of antibodies/ probes

**Antibody/ probe**	**Source**	**Dilution**
CD5 (SP19)	Cell Marque Co.,	Predilute
CD10 (56C6)	Cell Marque Co.,	Predilute
CD20 (L26)	Ventana, Arizona, USA	Predilute
CD23 (1B12)	Cell Marque Co.	Predilute
CD43 (L60)	Ventana, Arizona, USA	Predilute
Cyclin D1(SP4)	Medicorp, Montreal, Canada	1;50
BCL-2 (124)	DAKO, Mississauga, Canada	1:40
Kappa	DAKO, Mississauga, Canada	1:20,000
Lambda	DAKO, Mississauga, Canada	1:20,000
EBER (RNA probe)	Ventana, Arizona, USA	Predilute

### Immunophenotyping by flow cytometry

Mononuclear cells from fresh peripheral blood were separated by density-gradient centrifugation and were characterized using four-color immunostaining by a FACSCalibur flow cytometer (Becton, Dickinson and Company, San Jose, CA) and Cell Quest software (Becton, Dickinson and Company, San Jose, CA). The following monoclonal antibodies were used: CD45-allophycocyanin (APC), CD5-phycoerythrin (PE), CD19-peridinin cholorophyll protein (PerCP), CD20-fluorescin isothiocyanate (FITC), CD22-FITC, CD23-FITC, CD38-PE, CD11c-PE, CD10-PE, FMC7-FITC, anti-kappa chain-FITC, anti-lambda chain-PE. CD23 and FMC7 were obtained from Beckman Coulter (Miami, FL), and the rest of the monoclonal antibodies were purchased from Becton, Dickinson and Company.

### Molecular analysis

DNA from paraffin-embedded spleen sections was extracted using the commercially available DNA Blood Mini Kit (Qiagen Inc., Mississauga, ON, Canada). B cell clonality was assessed using nested PCR with consensus primers for the variable and joining regions as described by Reed et al. [11]. This approach is reported to detect 83% of immunoglobulin heavy chain gene rearrangements. T-cell clonality was similarly assessed using PCR-based method for T-cell receptor γ gene rearrangement described by Diss et al. [12]. Two reactions with primers VgI and VgIII/IV and Jg1/2 are reported to detect 80% of T-cell gamma gene rearrangements. A nested PCR was also used to detect *BCL2/IG* gene rearrangement targeting the usual breakpoint regions of the t(14;18).

## Results

**Figure 1:**
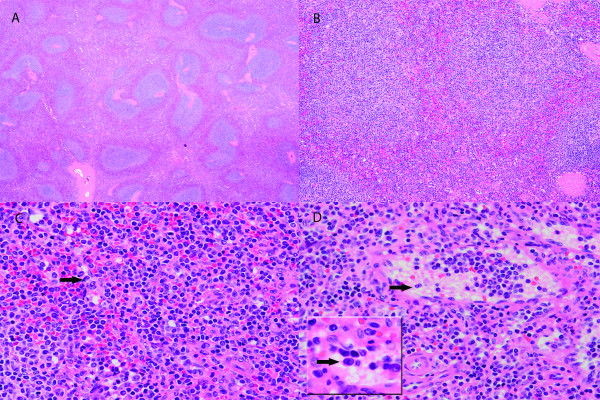
**Histological features of the spleen.** Images of the H&E stained spleen sections at 20x (**A**), 100x (**B**) and 400x (**C, D**) magnification showing expansion of the white pulp nodules and significant infiltration of the red pulp by small mature lymphocytes with minimal cytologic atypia. Occasional binucleated lymphocytes are noted in the splenic sinusoids indicated by black arrows (C, D and D inset).

Grossly, the spleen was enlarged, weighing 519 grams and measuring 16 x 11 x 6.5 cm. The splenic capsule was well preserved. Histologically, the spleen architecture was altered with expansion of the white pulp nodules by small mature lymphocytes with no prominent germinal center formations identified (Figure 1 - A, B). The white pulp nodules in some areas demonstrated fusion but no apparent or distinct marginal zones were present. There was also a massive infiltration of red pulp by similar-appearing small lymphocytes that were located both within the sinusoids and splenic cords (Figure 1 - C, D). Occasional binucleated lymphocytes were noted in the splenic sinusoids (black arrows in Figure 1 - C, D and inset). No large transformed lymphoid cells were identified and plasma cells were not prominent.

**Figure 2:**
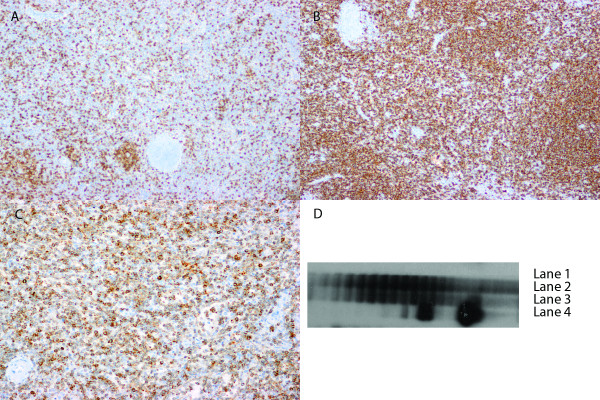
**Immunohistochemical features of the spleen.** Only few scattered T lymphocytes are seen as highlighted by CD3 stain (**A**, magnification 100x). Majority of lymphocytes in the white and red pulp are CD20 positive (**B**, magnification 100x) and BCL-2 positive (**C**, magnification 200x) B cells. PCR for *IgH* gene rearrangement performed on spleen paraffin-embedded sections reveals polyclonal pattern with no clonal rearrangements detected (**D**) (Lane 1 - no DNA; Lane 2 - patient; Lane 3 - negative control; Lane 4 - positive control).

Immunohistochemical analysis demonstrated massive amount of CD20+/ BCL-2+/ CD43+ B cells located both within the white pulp nodules as well as within the red pulp (CD20 and BCL-2 stains shown in Figure 2 - B and C respectively). These B cells were negative for CD10, CD23, CD5, and cyclin D1. Only few CD3+ T cells were present in the red and white pulp (Figure 2 - A). Scattered plasma cells were polytypic using Kappa and Lambda light chain stains and no monotypic restriction was identified within the B lymphocytes. No EBV was detected using EBER in situ hybridization.

Molecular analysis on paraffin- embedded spleen sample did not demonstrate clonal rearrangements of the immunoglobulin heavy chain genes (Figure 2 - D) or T-cell receptor gamma gene. No *BCL2* gene rearrangements were detected.

## Discussion

PPBL is an uncommon disorder characterized by an indolent and benign clinical course with persistent polyclonal B-cell lymphocytosis and circulating binucleated lymphocytes as well as increased polyclonal serum IgM. There are only rare reports of PPBL in association with malignant lymphoma or with secondary solid cancers [2,3,13]. Since the first description in 1982, about 200 cases have been reported in the literature.

The majority of the reports focus on the study of PPBL pathogenesis, which remains unclear. Although most frequently detected in smokers, PPBL is also occasionally observed in non-smokers. The association with viral infections, such as Epstein-Barr virus, is still a matter of debate [3,14-16]. The polyclonal B cells which are expanded in this disorder appear to be CD27+/IgM+/IgD + memory B cells, which may result from chronic antigenic stimulation [4,17]. PPBL is frequently linked with HLA-DR7 haplotype [18-20]. Cases of familial PPBL and the incidence of PPBL in monozygotic twins are suggestive of a strong genetic predisposition [21,22]. It is reasonable to speculate that the interaction of genetic predisposition with chronic antigenic stimulation may lead to the development of PPBL. Very recently, whole genome microarray expression analysis was done in 14 PPBL patients, which demonstrated over-expression of AP-1 transcription complex and downregulation of Fas-apoptotic and TGFβ pathway [5]. The polyclonality of the lymphocyte population evidenced by flow cytometry in this disorder is challenged by rare reports of clonal *IGHD-IGHJ* immunoglobulin rearrangement in patients otherwise meeting diagnostic criteria of PPBL [6,23]. These findings suggest that polyclonal expansion may be followed by the emergence of predominant clone in rare cases.

PPBL mimics malignant lymphoma morphologically. Variable amount of hallmark atypical lymphocytes are invariably present in peripheral circulation. Bone marrow changes in the PPBL patients described earlier demonstrate an interstitial, particularly intrasinusoidal B cells mimicking those seen in B-cell lymphoproliferative disorders especially in splenic marginal zone lymphoma [7]. However, the intravascular or intrasinusoidal pattern of the B cell distribution in the bone marrow is most likely a reflection of the peripheral lymphocytosis and the recirculating nature of the lymphocytes in this benign disorder. Mild splenomegaly is the most frequently reported physical finding, which is detected in about 10% of patients according to the largest case series [2]. Massive splenomegaly is rare among these patients. Our PPBL patient reported here manifested slowly progressive splenomegaly during six years of follow-up. Her spleen contained massive amount of CD20+/BCL-2+ B cells within the red and white pulp mimicking B-cell lymphoma. In their series of 5 patients Del Giudice *et al.* from Italy recently reported very similar findings to ours in three of their five PPBL patients who developed massive splenomegaly and underwent splenectomy [8]. The B cells present both in bone marrow and spleen show same immunophenotype including expression of BCL-2 [7,8]. In addition, the B cells in our patient were also positive for CD43. CD43 expression was not studied by Del Giudice *et al.* and CD43 was negative in the bone marrow reported by Feugier *et al.*[7]. Although expression of CD43 by B cells is often used as a marker in favor of a B-cell lymphoproliferative disorder, it has been recognized that CD43 can be expressed by B cells in benign conditions [24,25]. Except splenomegaly, no other abnormal physical or radiographic findings suggestive of malignant lymphoma transformation were detected in our patient and the five patients reported by Del Giudice *et al*. Therefore, the histological and immunophenotypic findings observed in the spleens of these PPBL patients are most likely a reflection of their underlying benign PPBL process.

PPBL also mimics lymphoma at cytogenetic and molecular level. The chromosomal abnormalities are frequently reported in PPBL patients. Isochromosome + i(3q) is the most common chromosomal abnormality and is present in 71% of cases when using the most sensitive fluorescence in situ hybridization (FISH) method [2]. Other less common chromosomal abnormalities include trisomy 3, premature chromosome condensation (PCC) and chromosome instability [2,9,10]. Among the above mentioned chromosomal abnormalities, trisomy 3 has been reported to be associated with marginal zone lymphomas (MZL) and mantle cell lymphoma (MCL) [26,27]. Cytogenetic studies on our patient using FISH were performed on the peripheral blood sample collected during one of the follow up visits and demonstrated no abnormalities of chromosome 3. Although not detected in our patient, *BCL2/IgH* gene rearrangements as seen in follicular lymphoma have been reported in some PPBL patients by using PCR technique [7,10,28].

## Conclusions

Our report adds to the extremely limited literature about the histopathologic features of PPBL; to our knowledge this is one of the first detailed descriptions of spleen pathology in a patient with PPBL. Both histological and immunohistochemical findings were misleading and mimicked B cell lymphoma. To avoid misdiagnosing this process as B-cell lymphoma, which may lead to unnecessary treatment, it is important to recognize the misleading histology and somewhat unusual phenotype, assess clonality and to be aware of the cytogenetic and molecular abnormalities that may be associated with this intriguing but benign entity.

## Ethical approval

This case report was based on the existing data, and the patients’ identification was kept confidential in this study. This case report does not meet definition of human or animal subject research by University and Medical Center Institutional Review Board of East Carolina University and University of Manitoba, and no ethical approval was necessary for this study.

## Competing interests

Both authors declare that they have no competing interests.

## Authors’ contributions

PS performed literature review, patient records review and drafted the manuscript. RJ conceived of the study, participated in its design and coordination, helped to draft and edited the manuscript and created the figures. PS and RJ were directly involved in the diagnosis and care of the patient. All authors read and approved the final manuscript.
